# Complexity of locomotion activities in an outside-of-the-lab wearable motion capture dataset

**DOI:** 10.3389/fbioe.2022.918939

**Published:** 2022-10-14

**Authors:** Abhishek Sharma, Eric Rombokas

**Affiliations:** ^1^ Department of Mechanical Engineering, University of Washington, Seattle, WA, United States; ^2^ Department of Electrical Engineering, University of Washington, Seattle, WA, United States

**Keywords:** complexity, wearable motion capture, out-of-the-lab datasets, gait analysis, real-world scenario, on-field movement analysis, human locomotion

## Abstract

Gait complexity is widely used to understand risk factors for injury, rehabilitation, the performance of assistive devices, and other matters of clinical interest. We analyze the complexity of out-of-the-lab locomotion activities *via* measures that have previously been used in gait analysis literature, as well as measures from other domains of data analysis. We categorize these broadly as quantifying either the intrinsic dimensionality, the variability, or the regularity, periodicity, or self-similarity of the data from a nonlinear dynamical systems perspective. We perform this analysis on a novel full-body motion capture dataset collected in out-of-the-lab conditions for a variety of indoor environments. This is a unique dataset with a large amount (over 24 h total) of data from participants behaving without low-level instructions in out-of-the-lab indoor environments. We show that reasonable complexity measures can yield surprising, and even profoundly contradictory, results. We suggest that future complexity analysis can use these guidelines to be more specific and intentional about what aspect of complexity a quantitative measure expresses. This will become more important as wearable motion capture technology increasingly allows for comparison of ecologically relevant behavior with lab-based measurements.

## 1 Introduction

Measurement of the complexity of motor output (Decker et al., 2010; Morrison and Newell, 2015) is a common and essential component of gait analysis. It can be used for basic science, providing a window into how the brain generates movement Ting and McKay (2007), performs sensation, and how neural control interacts with biomechanics (Duysens et al., 2013). It can also be used for clinical gait analysis, with real implications for prescription of interventions and functional classification e.g., (Steele et al., 2015a). For example, a decrease in motor output complexity might indicate a reduced ability to adapt to stresses (Peng et al., 1993; Amaral et al., 1998; Goldberger et al., 2002). According to this reasoning, decreased complexity could indicate a reduced capacity for rejection of variability Goldberger et al. (2002), or a deterioration of the complex human rhythms of movement associated with healthy function Decker et al. (2010). It might also serve as a tool to examine how well the current techniques used in control of assistive devices approximate the natural human gait. For example, it is well known that human gait exhibits variability (one measure of complexity) across strides due to several factors like environment or fatigue, while the control of assistive devices is often rigid and deterministic. Since different activities exhibit varying degrees of complexity, it may be that if a deterministic control technique works for one activity with low variability, it will not translate well to different, highly variable, activity. Thus, examining the variability (and more broadly, the complexity) of different activities, is needed.

In the past, gait datasets have been largely confined to in-the-lab environments. Most available gait data has been restricted to uncluttered level ground ambulation or walking on a treadmill. As a result, much of the analyses and conclusions about human gait are drawn from a limited context. For example, there are no previous studies that compare commonly recorded gait activities like forward walking in a straight line to daily unconstrained walking in public places in terms of their complexity. However, recent developments in wearable sensors have driven increased interest in measuring human movement under a more diverse set of activities and situations. This makes it possible to analyze and compare these activities with the most commonly analyzed activity: flat ground walking in a straight line.

It is actually not trivial to quantitatively measure and define the relative complexity of different activities (Morrison and Newell, 2015). From our natural experience of life, we understand that avoiding obstacles, navigating challenging terrain, or dealing with uncertainty in the environment should result in more complex movement. We also intuit that movement outside of a gait lab, in the presence of other people and a changing environment, should result in more complex movement. But what, precisely and quantitatively, does that mean? There are several reasonable quantitative measures of complexity that actually are measuring different aspects of the data, and *can be contradictory.*

In this manuscript, we attempt to define reasonable boundaries for these questions, and demonstrate some experiments and measurements that begin to answer them. Our goal is to contribute to a standard practice of gait complexity analysis, and especially comparison of different activities, as movement studies increasingly take place in more natural, unconstrained contexts. We present a multi-subject (See Table 1) full body kinematics dataset that captures diverse activities like forward walking, backward walking, side stepping, avoiding obstacles by stepping over them, navigating around obstacles in structured and controlled environments as well as unstructured and uncontrolled natural environments, and stair ascent and descent. We qualitatively and quantitatively compare these activities to straight-line forward walking.

**TABLE 1:** Activities and subject details.

Activities	# Subjects	Age (yrs)	Height (cm)
Forward walking	9 females; 11 males	26.2 ± 2.7	174 ± 10.9
Backward walking	4 females; 5 males	21.5 ± 2.4	173.4 ± 6.9
Sidestepping	4 females; 5 males	21.8 ± 2.2	172.8 ± 6.8
Classrooms and Atrium	12 females; 11 males	22.8 ± 2.7	171.2 ± 9.7

First, we provide background and context for complexity analysis in Section 2. We also present the potential contradictions in different complexity measures using a toy example. In Methods (Section 3), we describe the experiment, data analysis details, and quantitative outcome calculation methods. In Results and Discussion (Section 4) we present comparisons of the relative complexity of the different activities, and consider the importance of these outcomes, especially when different notions of complexity result in apparent differences. Finally, we discuss some limitations of our analysis.

## 2 Background: Complexity analysis

Previous analyses of complexity may be generally categorized as being inspired by three notions (Decker et al., 2010; Morrison and Newell, 2015): 1) dimensionality, 2) variability, and 3) nonlinear dynamics. Here we use measures from each of these. As we describe in the Results and Discussion, there can be important differences in the apparent complexity of gait depending on the specific measures being used.

### 2.1 Complexity in terms of dimensionality

This approach assumes that the greater the number of dimensions (degrees of freedom) required to describe the data, the greater the complexity of the data (Morrison and Newell, 2015). A common method used to capture dimensionality of the data is Principal Component Analysis (PCA). Dimensionality is defined as the number of principal components required to capture a certain level of variance in the data. The greater number of PCs required to explain the desired level of variance in the data, the greater the complexity of the data. There are a variety of other matrix factorization algorithms are used to identify underlying regularities or synergies in movement data (Steele et al., 2015b). As we have shown in other work, there are other advantageous nonlinear methods of identifying the underlying dimensionality (Portnova-Fahreeva et al., 2020; Boe et al., 2021). However, the most straightfoward method commonly used in the current gait literature is PCA (Morrison and Newell, 2015), so we will constrain ourselves to that measure for this analysis.

### 2.2 Complexity in terms of variability

An alternative way to measure complexity is to assess the amount of deviation in a signal. For example, the Standard Deviation (SD) or Coefficient of Variation are common measures that use this approach (Morrison and Newell, 2015). This allows the complexity of even very low-dimensional data to be quantified meaningfully. For multi-variate data the determinant of the covariance matrix, also known as Generalized Variance (Wilks, 1932), can be used a variance measure. Another measure of variability (GaitSD) has been proposed in (Sangeux et al., 2016), to measure the variability of gait waveforms across strides. Larger variability implies greater complexity under these definitions.

### 2.3 Complexity in terms of non-linear dynamics

Tools from non-linear dynamical system theory have been used to measure the regularity and periodicity of gait signals across time (Decker et al., 2010). describes two kinds of analyses: State space examination and self-similarity evaluation, used to assess gait complexity.

State-space examination is done using the Largest Lyapunov Exponent (LyE) and Correlation Dimension. The LyE measures the average exponential rate of separation of neighboring trajectories of the attractor, while Correlation Dimension is a measure of the fractal dimension of the attractor. A positive LyE indicates aperiodic signals while a negative or zero LyE are associated with periodic signals. Random data are generally characterized by a large Correlation Dimension and LyE values while deterministic (periodic or chaotic) data exhibit smaller values.

Self-similarity evaluation is done to examine the presence of repeating patterns in the gait signal. Entropy based measures like approximate entropy (ApEn), sample entropy (SampEn), detrended fluctuation analysis (DFA) and multiscale entropy (MSE) are used to this end (Costa et al., 2005).

### 2.4 Contradictions in measures of complexity

**FIGURE 1:**
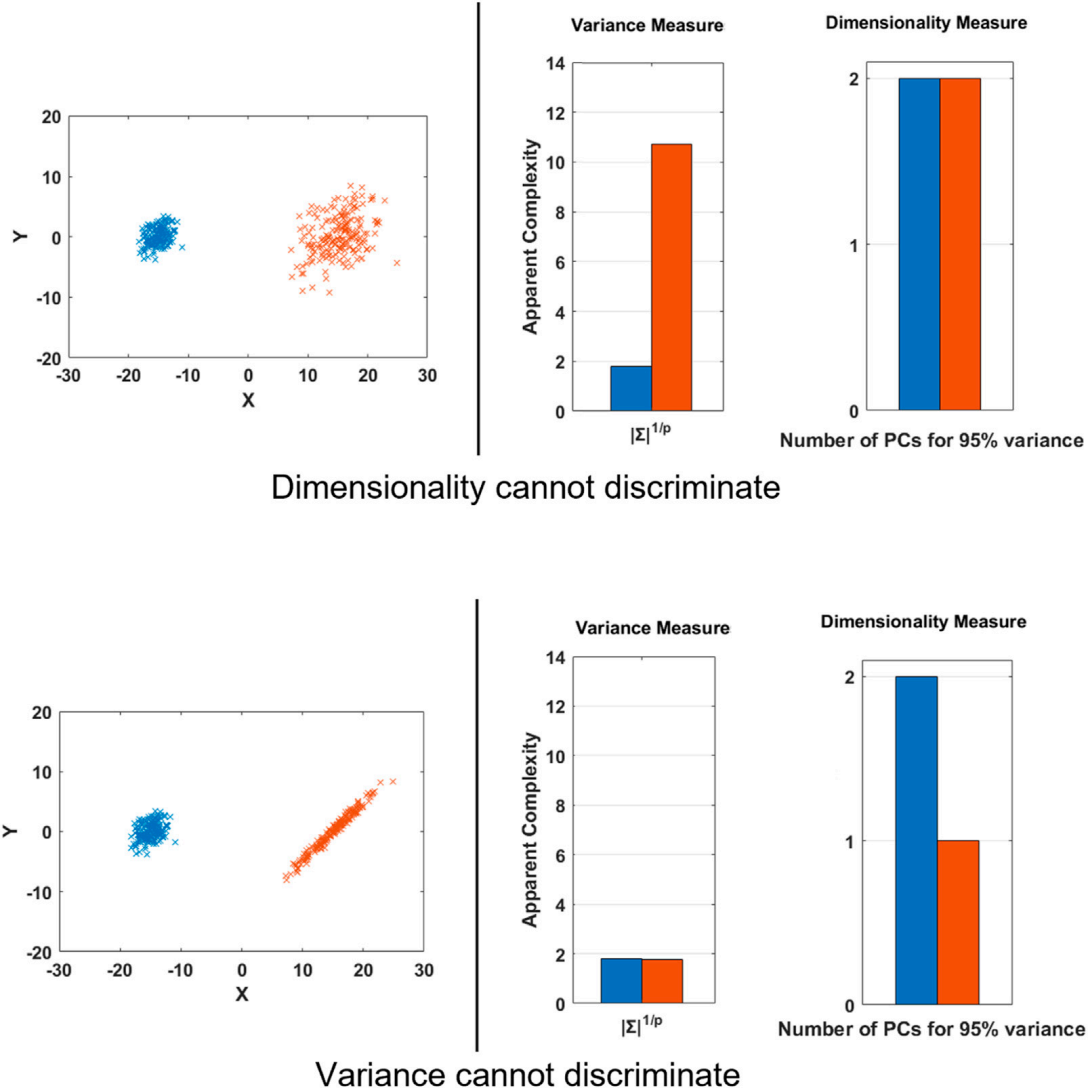
Toy Example to demonstrate some cases when different measures of complexity can fail to discriminate two distinct datasets and lead to contradictory outcomes. This example deals with only variance and dimensionality, but similar parallels exist for the other measures of complexity, such as stability from a nonlinear dynamics perspective.

These are all reasonable, but potentially contradictory, quantitative measures of complexity because they are measuring different characteristics of the data. We can understand this from the following 2D toy example in Figure 1 as follows: In the first row, we see two Gaussian data clouds which we can imagine as being generated by two different activities. Consider if we define variance of the data as the measure of complexity. We could reasonably use Generalized Variance for multi-dimensional data (Wilks, 1932), which is defined as the determinant of the covariance matrix (Σ). We would rank the red cloud to have greater complexity, because the red cloud implies people need to attain a broader range of distinct states with their body. On the other hand, if we use dimensionality as the measure of complexity, we would not be able to distinguish between the two activities, as both equally employ the 2 available degrees of freedom.

In the second row, we see that a dimensionality measure would rank the blue activity to be more complex, since the red activity seems to be generated by a single independent factor, while the generalized variance would rank both the activity clouds to be of similar complexity.

These examples highlight that we need to exercise caution when discussing complexity. Although it appears to be a concrete and quantitative concept, it is necessary to be more specific about what kind of complexity we are measuring. In the toy example, simple visualization of the data helps to provide an intuitive grounding, but as we analyze time series data from many sensors simultaneously, we cannot rely on intuition. In the remainder of this manuscript, we will demonstrate this concretely using five standard complexity measures.

## 3 Methods

### 3.1 Experiments and subjects

For each data collection session, the subject was briefed about the experiment and informed consent was obtained. All activities were approved by the Institutional Review Board at University of Washington. The entire dataset will be made available on a public repository (https://github.com/abs711/The-way-of-the-future) and more details about the data are presented in (Sharma et al., 2022) Subjects’ joint kinematics were recorded using an Xsens Awinda full body motion capture system (*Xsens Technologies, Enschede, Netherlands*), consisting of 17 body-worn inertial measurement units placed at each segment of the limbs, as well as sternum, sacrum, shoulder scapula, and forehead. After a system specified n-pose calibration, the software provides joint kinematics in a 3D environment. All angles are in 1 × 3 Euler representation of the joint angle vector (x, y, z) in degrees, calculated using the Euler sequence ZXY using the International Society of Biomechanics standard joint angle coordinate system (Wu et al., 2002). Data were sampled at 60 Hz, from a total of 22 joints in 3 anatomical planes (sagittal, frontal, transverse) for each trial. The kinematics data were reprocessed using the ‘HD’ processing feature, provided by the manufacturer for offline use, to enhance quality and remove noise (Myn et al., 2015).

In this manuscript we limit the complexity analysis to kinematics data from only the lower limb joints: hip, knee and ankle from sagittal, transverse, and frontal planes for both the limbs. Thus, a total of 3 anatomical planes from 6 lower limb joints were used in our analysis i.e., 18 degrees of freedom.

**FIGURE 2:**
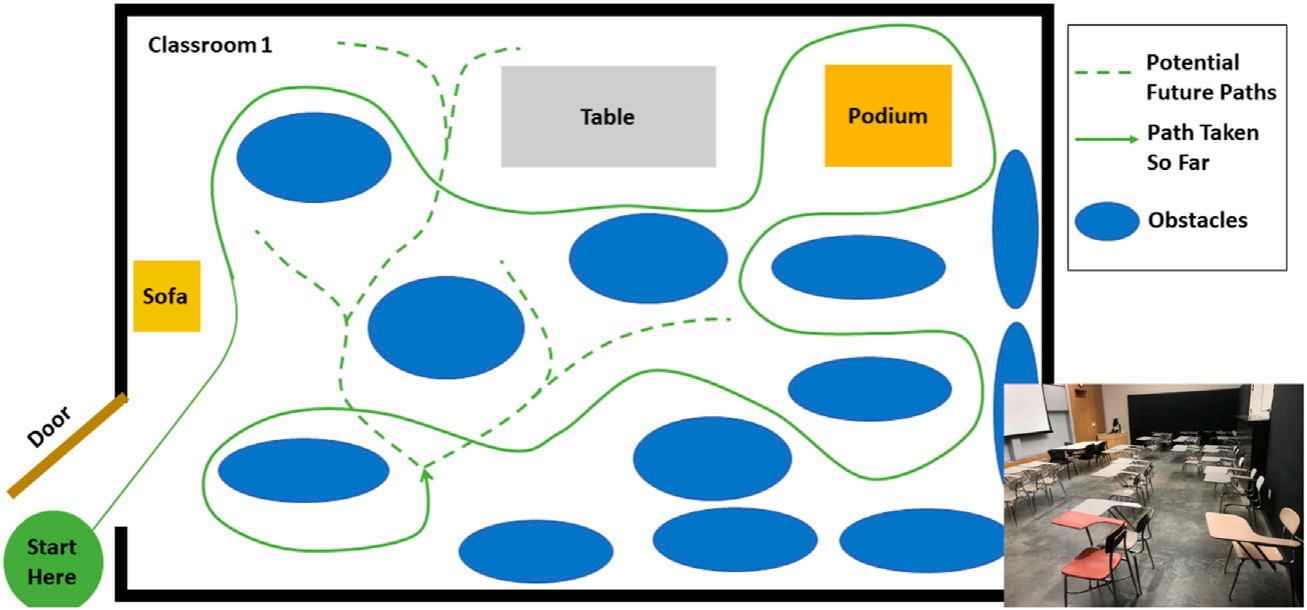
Classroom: Architecture of one of the classrooms. The arrangement of obstacles was not controlled and varies across subjects. The subject walked at self-selected speed and along self-selected path, The experimenter directed the subject to change their path only if the subject repeated the same path more than 2 times.

**FIGURE 3:**
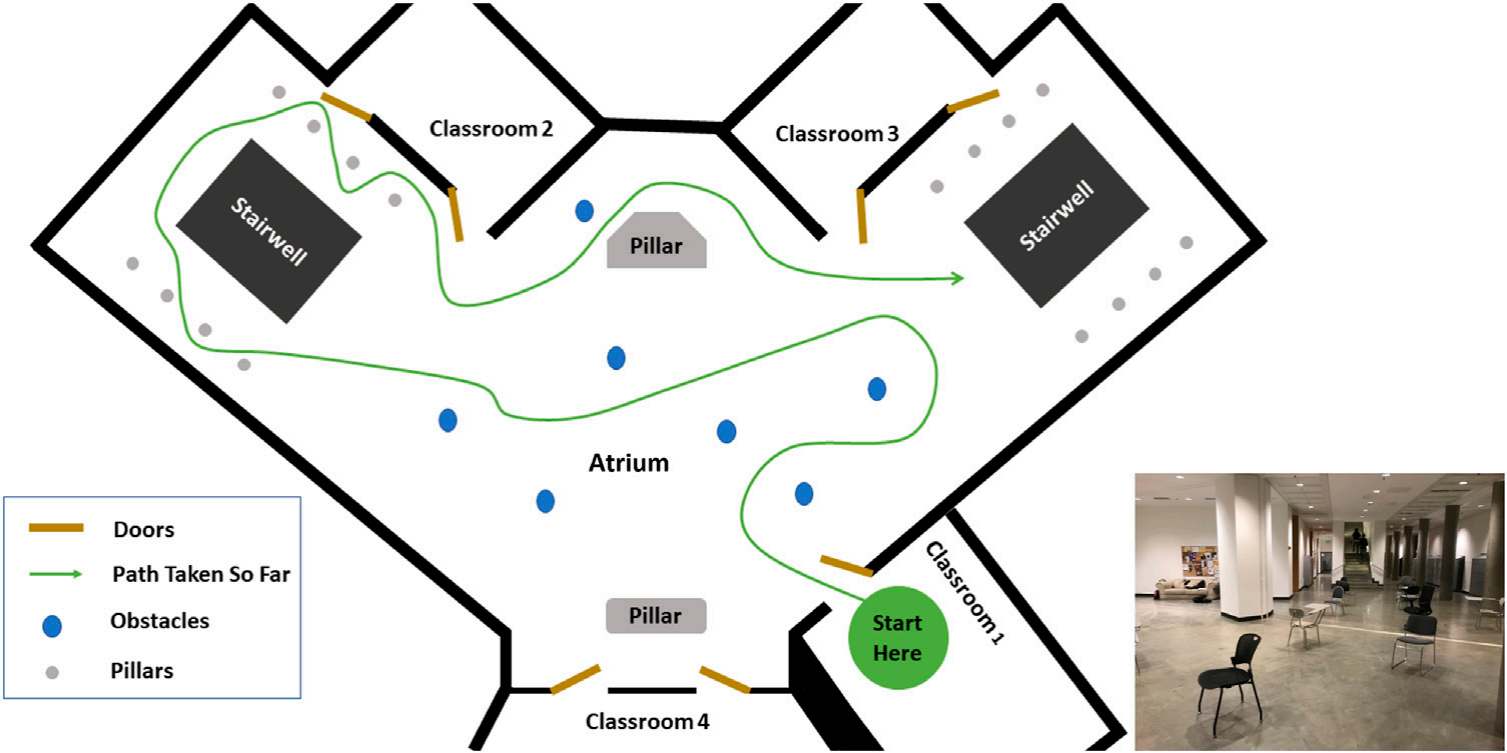
Atrium: Architecture of the Atrium. The arrangement of obstacles was not controlled and varies across subjects. The subject walked at self-selected speed and along self-selected path, The experimenter directed the subject to change their path only if the subject repeated the same path more than 2 times.

Subjects ambulated in a variety of ways, including walking, sidestepping without crossing legs, navigating through obstacles, making turns, etc. as they deemed necessary in order to navigate the environment. Their speed was self-selected and their path around obstacles was not instructed. The movement was performed outside of a laboratory, in the corridors, indoor rooms and atrium of a building. The architecture for one of the classrooms and the atrium is shown in Figures 2, 3. The dataset was manually parsed into six activities for analysis. The activities that were parsed out for complexity analysis are: 1) **Forward walking** (straight line), 2) **Backward walking** (straight line), 3) **Left sidestepping**, 4) **Right sidestepping**, 5) **Navigating in classrooms**, and 6) **Navigating in an atrium**.

The numbers of participants and demographic information for each of the activities are shown in Table 1.

### 3.2 Data analysis

In the Background: Complexity Analysis section above, we described three major notions that can be used to analyze complexity: Dimensionality, Variability, and Nonlinear Dynamics. We used measures related to these notions as described below, to analyze the complexity of activities.

#### 3.2.1 Dimensionality

The dimensionality of an activity is defined as the number of PCA principal components required to explain 95% variance in the activity (*N*_95%_). We used the function ’pca’ from the Statistics and Machine Learning Toolbox. MATLAB 2020b for the analysis. *N*_95%_ was computed using the matrix of 18 dimensional time series from each trial. The mean and standard deviation of *N*_95%_ across trials are reported for each activity.

#### 3.2.2 Variability

We examined variability according to two different measures. The first is the Determinant of the data covariance matrix. It is not in standard use for human movement analysis, but it is a longstanding way to quantify variance in multidimensional data (Wilks, 1932). The second measure is GaitSD, which measures how variable the gait cycles are from one another (Sangeux et al., 2016). GaitSD is described in Eq. 1.

$$\begin{array}{cc} X_{ij} & =i^{th}\text{ gait cycle defined over T time instances, T}=\;101\\ X_{j} & =\frac{1}{N}\overset{N}{\underset{i=1}{\sum }}X_{ij}\text{ , }N=\text{number of gait cycles}\\ GVSD^{2} & =\frac{\sum_{j=1}^{T}\sum_{i=1}^{N}{\left(X_{ij}-X_{j}\right)}^{2}}{T\left(N-1\right)}\\ GaitSD & =\sqrt{\frac{1}{p}\overset{p}{\underset{k=1}{\sum }}GVSD_{k}^{2}}\text{ , }\text{p }\left(\text{number of joints}\right)=18. \end{array}$$ (1)

Gait cycles were determined using the foot contact data provided by Xsens, and all the joint angles were time normalized to 101 points using the MATLAB command- *’interp1’*.

#### 3.2.3 Nonlinear dynamics

Following methods from (Decker et al., 2010; Busa and van Emmerik, 2016), we used the Largest Lyapunov Exponent (LyE), and Multiscale Entropy (MSE). These measures were calculated using ankle, knee, and hip kinematics in the sagittal plane.

To calculate the LyE, we first reconstructed the state space from one dimensional time series (sagittal ankle, knee, and hip separately), using Takens’ theorem (Noakes, 1991). The delay for reconstruction was estimated using Average Mutual Information (AMI) (Fraser and Swinney, 1986). It was set to be the first local minimum of AMI. Embedding dimensions were determined using Global False Nearest Neighbors (GFNN) analysis (Kennel et al., 1992). Embedding dimension was set to the minimum value that satisfied percent false nearest neighbour less than 10%. LyE were then determined using MATLAB’s Predictive Maintenance Toolbox. The package calculates LyE using the algorithm developed by (Rosenstein et al., 1993).

Multiscale Entropy is a way to analyze the self-similarity of a one dimensional time series. There are multiple ways to calculate MSE (Humeau-Heurtier, 2015), but here we use a robust variant, Composite multiscale Entropy (CMSE), proposed in (Wu et al., 2013). The Complexity Index (CI) is defined in Eq. 2. *m* and *r* were chosen as 2 and 0.2 respectively in accordance with (Bisi et al., 2018) and values of *τ* ranged from 1 to 20.

$$\begin{array}{cc} CI & =\overset{N}{\underset{\tau =1}{\sum }}CMSE\left(x,\tau ,m,r\right)\\ \tau & =\text{time scale index, }\text{N }\left(\text{Total number of time scales}\right)=20 \end{array}$$ (2)

## 4 Results and discussion

**FIGURE 4:**
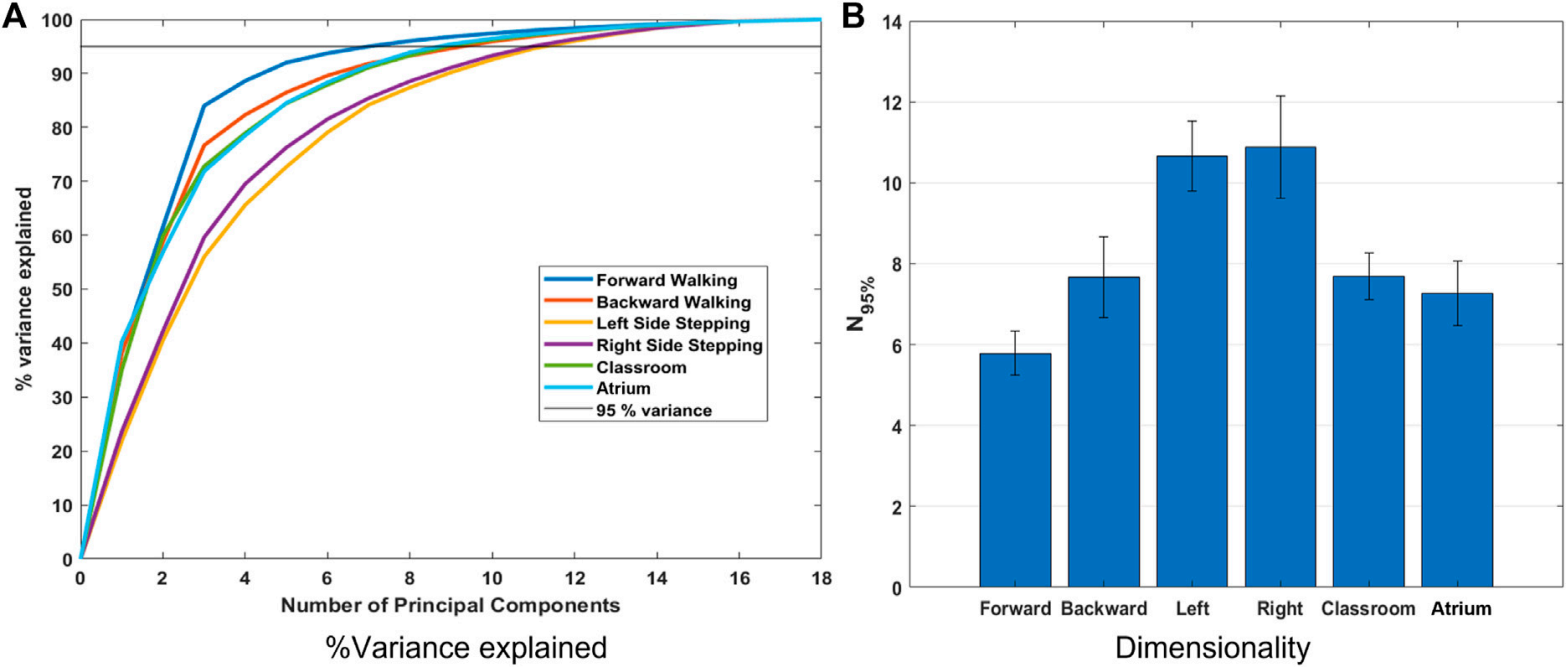
**(A)** Percent variance accounted for by each principal component and the sum of the first n principal components (line plots), for different activities. These were calculated using the data from all subjects. **(B)** **N**_**95%**_ values for all the activities. ***N***_**95%**_ is the number of principal components required to explain 95% variance in the data from each subject. *N*_95%_ indicates all other activities have higher dimensionality, and therefore complexity, than forward walking. Surprisingly, this metric indicates that left and right sidestepping are more complex than walking in a natural environment.

**FIGURE 5:**
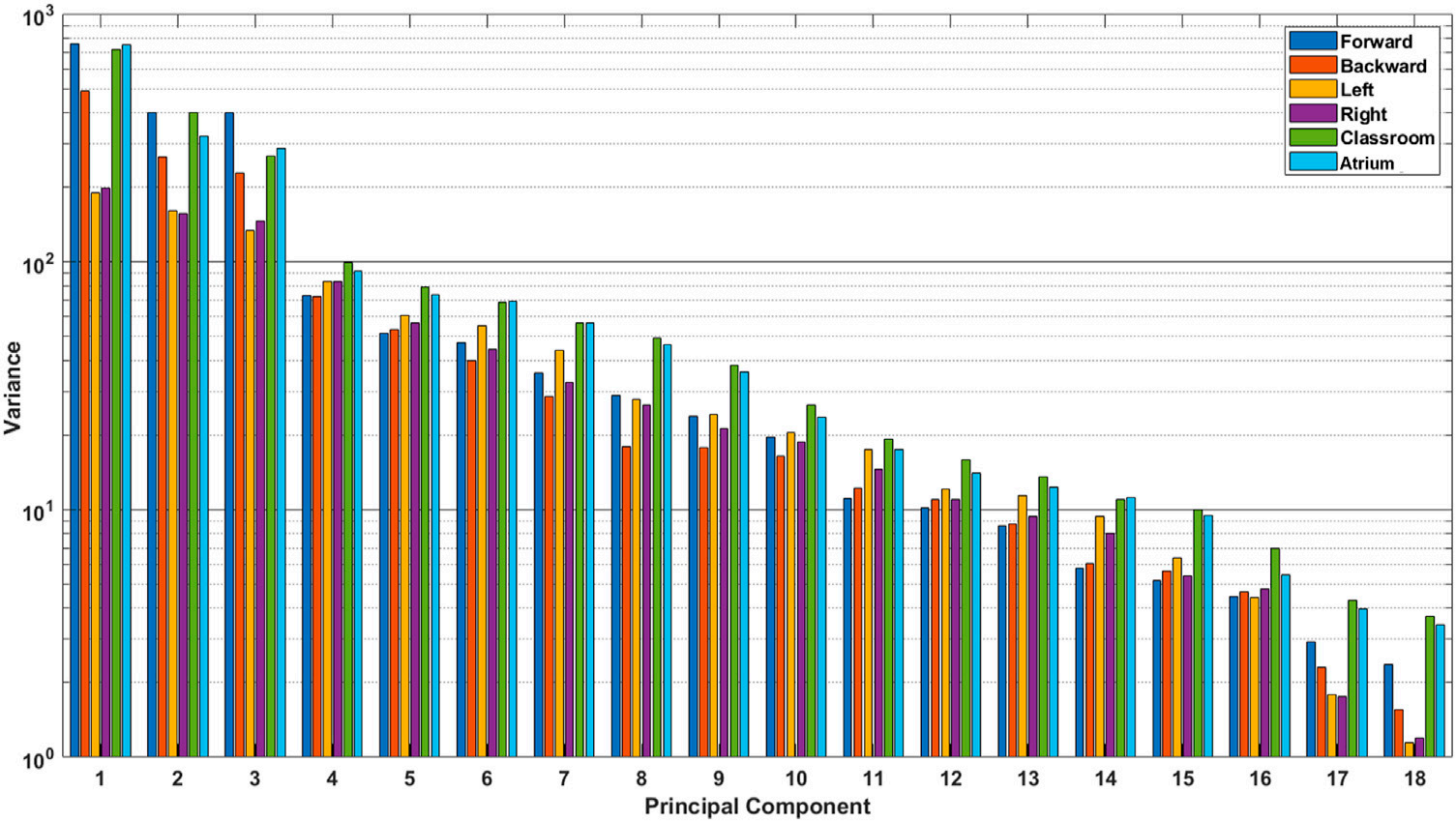
Absolute variance accounted for by each principal component, for different activities. These were calculated using the data from all subjects. We see that the last few principal components for Classroom and Atrium show considerably larger amount of variance than sidestepping, even though they are ignored by PCA when measuring dimensionality (see Figure 4).

**FIGURE 6:**
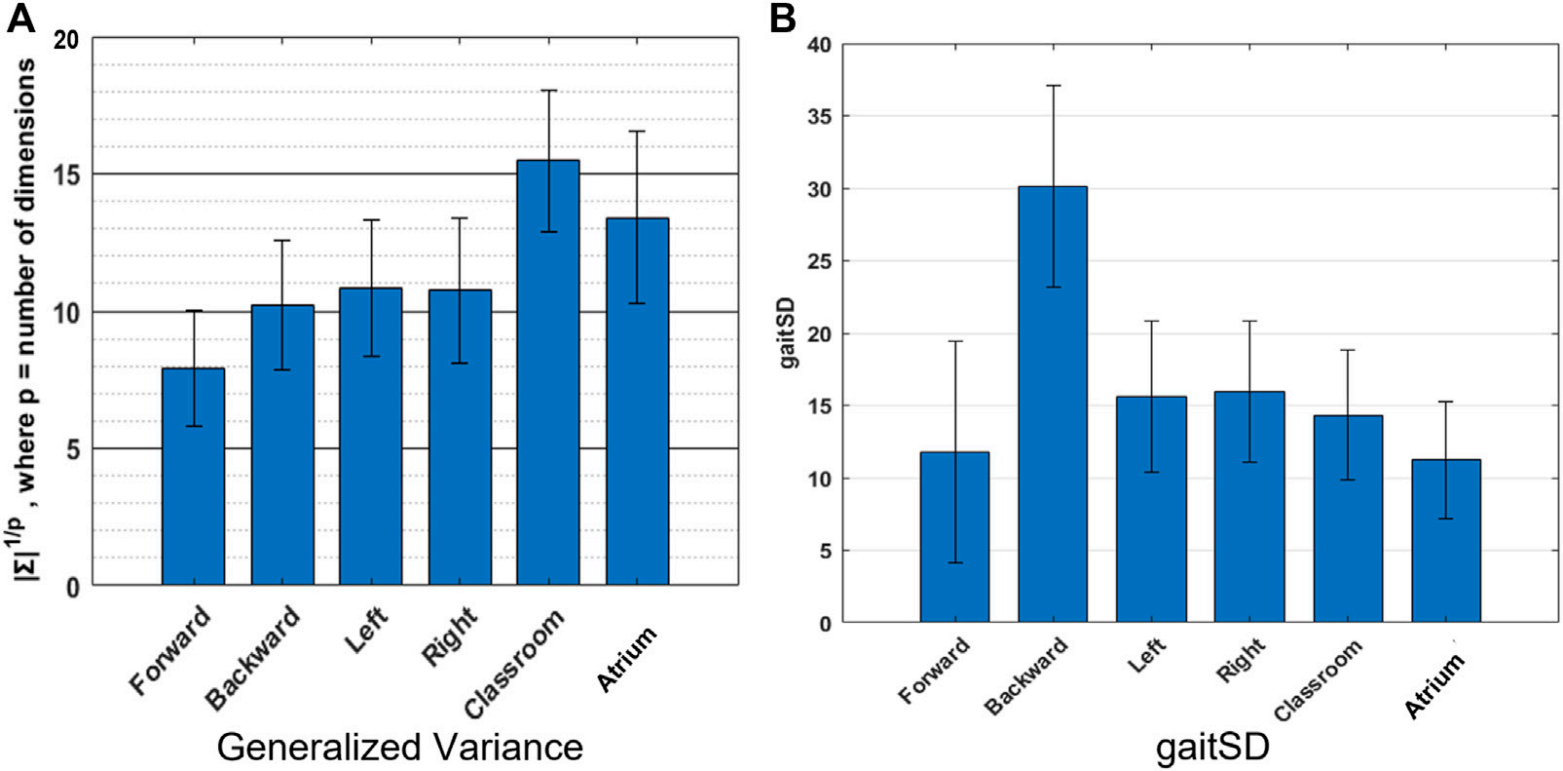
Variability: We use two different measures of variability-**(A)** Generalized Variance (geometric mean of the variances along the Principal Components) which measures the spread of the multi-dimensional data. Walking in classroom exhibits greater complexity in the joint angles, than other activties according to this metric, **(B)** GaitSD which measures variability of gait kinematics across strides, ranks backward walking to be of greatest complexities. The values reported are inter-subject mean and standard deviation.

**FIGURE 7:**
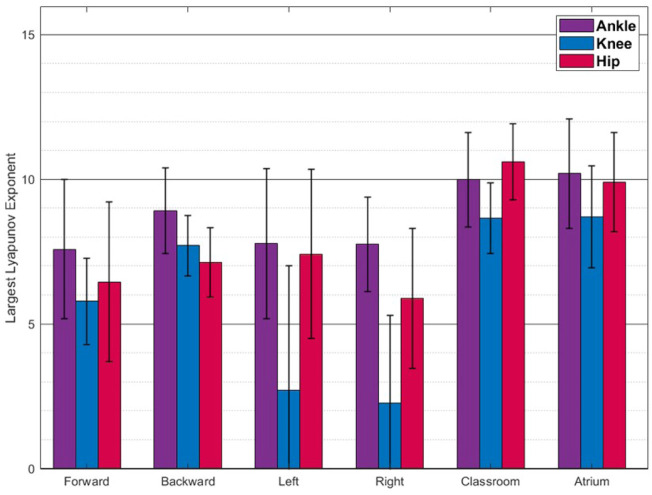
Largest Lyapunov Exponent(Mean **±** SD) The values reported are mean and standard deviation across the trials from all the subjects. Walking in classrooms and atrium shows greater complexity than other activities.

**FIGURE 8:**
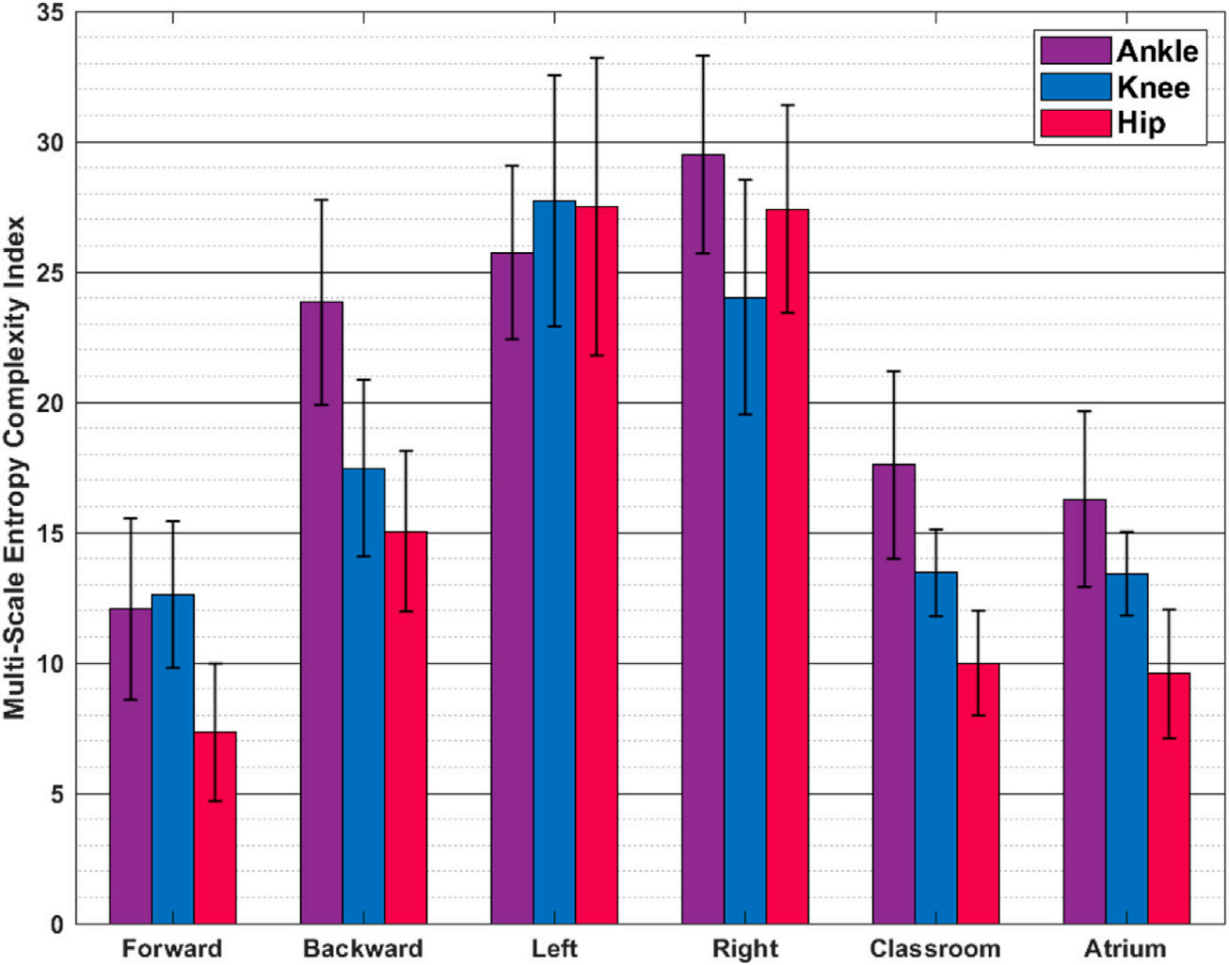
Multiscale Entropy (Mean **±** SD) The values reported are mean and standard deviation across the trials from all the subjects. Sidestepping shows greater irregularity and thus complexity, than other activities.

For each of the activities, we calculated the complexity measures of Dimensionality (Figures 4, 5), Variability (Figure 6), and Nonlinear Dynamics (Figures 7, 8). For each of these, we report the relative complexity of the activities and discuss when the results are contradictory or unexpected.

### 4.1 Dimensionality: Sidestepping is the most complex activity?

Figure 4A shows variance explained across subjects, from PCA of Lower Limb (18 dof) for the different activities. The number of principal components required to explain 95% of the variance (*N*_95%_) is shown in Figure 4B. We observe that left and right sidestepping require the most components to explain the variance, while forward walking requires the fewest. Complexity analysis in terms of dimensionality as measured by PCA, then, concludes that sidestepping is the most complex activity while forward straight line walking is the least.

Dimensionality is appealing as a measure of complexity because it aligns with the intuition that a “more complex” task should require more independence among its degrees of freedom. Dimensionality has been successfully used in gait analysis and has aligned with clinical notions of mobility and the scientific notion of synergies (Latash et al., 2007; Rombokas et al., 2012; Steele et al., 2015a). However, in this study we show that using PCA and ”variance accounted for” yields counterintuitive results. Although forward walking is measured as least complex, left and right sidestepping arise as the most complex, while navigating freely amongst challenging obstacles, as in the classroom activity, is measured as less complex than unobstructed sidestepping.

This result is surprising because from our experience of life, we understand that avoiding obstacles, navigating challenging terrain, dealing with uncertainty in the environment, etc. should result in more complex movement in Classrooms and Atrium. It should require us to use more degrees of freedom to navigate. This result can be interpreted in two mutually exclusive ways: 1) Even though sidestepping and backward walking are expected to be highly repetitious, they are less practiced, and thus show less coordination between joints. Thus, the data has more degrees of freedom than expected. 2) Alternatively, the result could be interpreted to indicate that PCA should not be used to measure and compare dimensionality when the two datasets have different overall absolute variance (See Figure 5). For example from Figure 4B, we see that Sidestepping has a dimensionality of approximately 11. Now, from Figure 5, we see that 11^*th*^ PC for Classrooms and Atrium has greater variance that for Sidestepping activities, but is ignored when 95% variance is used as the criterion to decide the dimensionality of data. This highlights the need for further examination of our intuition about the complexity of locomotion activities, and to be aware of these issues when using PCA for measuring the dimensionality of activities.

### 4.2 Variability: Classroom walking or backward walking is the most complex activity?

Figure 6A shows the generalized variance for the different activities. Forward walking shows the smallest generalized variance indicating tighter coordination of joints, while Classroom shows the largest value, indicating more variability in joint angles and less coordination amongst them. Figure 6B shows that backward walking has the largest GaitSD, indicating greater stride to stride variability of joint kinematics.

Variability is a perfectly reasonable way to quantify the complexity of data. While PCA uses variance and covariance to measure complexity, it only looks at how variance is distributed across different dimensions i.e. relative (or percent) variance. It can be instructive to look at absolute variance as well. Here we use generalized variance and GaitSD to measure variability in two distinct ways. Generalized variance is a measure of how much volume in the state space is occupied by a given activity. In other words how many different configurations of joints are achieved by a given activity. Navigating the Classroom and the Atrium must be expected to show greater generalized variance than other repetitious activities, because they require extemporaneous movements to avoid obstacles, change directions, etc. Generalized Variance comes out to be highest for those activities, matching our expectation.

GaitSD measures the variability of joint angles across gait cycles. It is not sensitive to the amplitude of joint angles (and thus the volume occupied in the joint-space) but instead the deviations at different phases in a gait cycle from the mean gait cycle. In other words, trying to do a repetitious activity but failing to do it exactly would have a greater GaitSD value than doing many kinds of movements but with more precision. This might explain why backward walking has a greater GaitSD value than other activities. Backward walking is presumably less practised in daily life than the other activities. Sidestepping also shows slightly higher values than other regularly practised activities like forward walking, walking in the classrooms and atrium.

This highlights that variability can be measured in different ways but more importantly the different measures need not agree. GaitSD, a measure of gait consistency, rates backward walking to have almost twice the amount of gait variability than unrestricted classroom walking. Further examination is required to understand the mechanisms leading to this observation, because naively we would expect unrestricted classroom and atrium walking to have greater variability than backward walking which is expected to be repetitious.

### 4.3 Nonlinear dynamics: Classroom or sidestepping is the most complex activity?

Figure 7 shows the LyEs for different activities. For the most part, the activities show positive LyE values indicating non-periodic gait signals. Classroom and Atrium show largest LyE of all the activities, across all the joints. For the knee joint, LyE of sidestepping for some subjects are negative or close to 0, while other subjects have large positive LyE (close to 6). This might be an artifact of noise in the data and needs more investigation. Figure 8 shows the analysis of ankle, knee and hip joint trajectories, using MSE, computed using sample entropy (*S*_*E*_) over 20 time-scales.

Human gait can be modelled as a dynamical system. Non-linearity in dynamical systems leads to different kinds of complexities than the ones we analyzed above. This has to do with periodicity, regularity and predictability of temporal dynamics of the system. We used LyE and Multi-scale Entropy to analyze complexity from this point of view.

LyE measures how quickly neighbouring trajectories in the dynamical system converge (negative values) or diverge (positive values). Larger positive values indicate faster divergence and thus lesser predictability of gait further into the future. Since, Classroom and Atrium exhibit largest values, they should be expected to be less predictable. This is expected because navigating obstacles would require significant deviation of gait from the immediate history, thus less predictability into the future.

Multi-Scale Entropy measures how many repeating patterns are there in a signal over different time-scales. Intuitively, it measures the regularity, or predictability, of a signal. We see that unusual activities i.e. sidestepping and backward walking show more irregularity in gait than more common activities i.e. forward walking, Classroom and Atrium. This might be a result of lack of practice in sidestepping and backward walking.

Once again we find major disagreement between the two measures used in this analysis, highlighting contradictions between different types of complexities in temporal dynamics.

### 4.4 Complexity cannot be defined as a unitary concept

**FIGURE 9:**
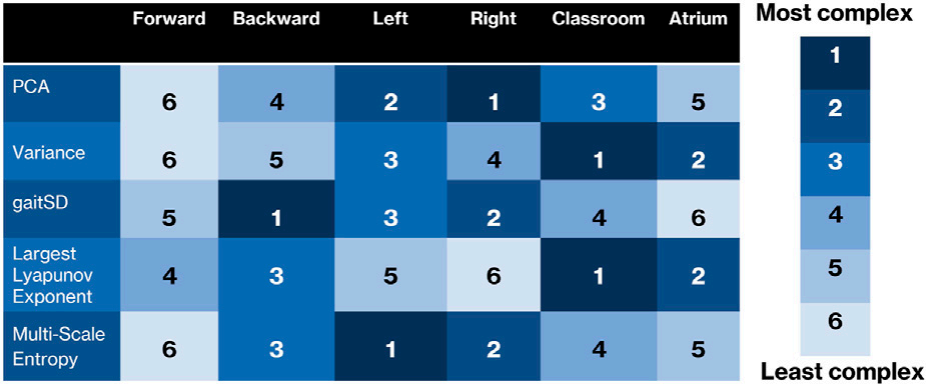
Overview of how each complexity measure ranks the six activities. While there are some similarities, it can be seen that each measure is sensitive to different characteristics of the complexity of the data, and that many of the results are surprising or counterintuitive.

These results demonstrate that there are several ways to measure different aspects of data complexity. These measures often do not rank activities similarly. For example, dimensionality as measured using PCA ranks sidestepping to be the most complex, but gait cycle variability as measured using GaitSD ranks backward walking to be the most complex, while divergent nonlinear dynamics as measured using LyE ranks atrium as the most complex. Looking forward for practitioners of movement analysis, *complexity* should probably be avoided as a single concept in favor of specific measures. For example, when we use PCA analysis, we should state that we are measuring degrees of freedom, not accounting for the scale of the variance. We summarize the rankings of complexity in Figure 9. As can be seen, no column, corresponding to activities, is agreed upon in complexity ranking by the different methods.

### 4.5 Forward walking is the least complex activity

Most of the measures agreed on forward walking being the simplest activity. Although GaitSD and LyE did not strictly rank it as the least complex, it is very close, as can be seen in Figures 6, 7. This is expected since forward walking is highly practiced and repetitious, and does not involve deviations to account for obstacles.

### 4.6 Practical recommendations

• PCA ranks sidestepping to be more complex and backward walking to be as complex as walking around obstacles in classrooms and atrium. This is counterintuitive. On further analysis, we found that sidestepping does not necessarily have more variance in the last PCs than classroom and atrium, as can be seen from Figure 5. This can be understood from the 2D toy example, as shown in Figure 1, bottom row. As can be seen, even if the minor principal component has the same variance for both blue and red clouds, PCA would rank the blue cloud to be more complex than red cloud, because it ignores the absolute variance and only accounts for relative variance. Thus, we need to account for absolute variance, before we use PCA to rank the dimensionality of different activities. To measure the absolute variance, we recommend that researchers use Generalized Variance.
• Usually, in the gait literature, variance is used to analyze one-dimensional signals. In our analysis, we used Generalized Variance as a measure of absolute variance for multi-dimensional data. We found that the resulting complexity ranking of the activities aligned well with our expectations. Thus, we recommend using Generalized Variance to measure the scale of the data.
• In our analysis, we found that Largest Lyapunov Exponent values to be quite different from (Buzzi et al., 2003). This could be attributed to sensitivity of the measure to noise in the data or the length of the data. In addition, computation of Largest Lyapunov Exponent assumes a time-invariant and autonomous dynamical system. Thus, we recommend against the use of the measure, unless the accompanying assumptions are tested for.
• In the gait literature, complexity is an umbrella term that measures different aspects of the data-dimensionality, variance and nonlinear dynamics. Since these measures do not always agree, we recommend against the usage of the term ‘complexity‘ and instead using the terms that emphasize the metric being used e.g. dimensionality.

## 5 Limitations

Dimensionality, as a concept used in mathematics, is much broader than we are using it here for gait analysis. For example, dimensionality can be defined as the number of Euclidean dimensions, topological dimensions (Suárez, 1994), fractal dimensions, etc. We only use number of principal components as it has a precedent in gait analysis. Even in terms of integer dimensions or degrees of freedom, other dimentionality reduction techniques like autoencoders could be used to estimate dimensionality (Portnova-Fahreeva et al., 2020; Boe et al., 2021).

Calculation of the Largest Lyapunov exponent requires the assumption that the system is *autonomous,* and time invariant (Sato et al., 1987; Rosenstein et al., 1993). This assumption could be broken by learning effects, fatigue, etc. Additionally the Largest Lyapunov exponent requires large amounts of data to be confidently calculated. So, care must be taken to ensure that adequate data sizes are used. It has been shown that accurate Lyapunov dimension calculation requires hundreds of gait cycles, and can be sensitive to preprocessing choices, such as using a fixed number of strides or a fixed number of data points (Hussain et al., 2020). When comparing activities that have very different total amounts of data, or different standards for preprocessing, care must be taken for this measure to be meaningful. This factor is not limited to Lyapunov dimension for gait; some measures, such as those used in heart rate variability estimation, have been shown to require small data sizes, while others require more data for robust estimation (Chou et al., 2021).

Since the data collection process is time consuming, any particular participant could not perform all of the different activities. While there is no missing data from any particular participant, each performed only a subset of the possible activities, as shown in Figure 1. As a result, the analyses we present here cannot account for individual differences in complexity. Individual gait characteristics could be practically important, for example in designing assistive devices, and should be accounted for also.

The data were also measured for a narrow age range of young people indoors, in an experimental session. We anticipate that their movement was more reflective of their natural patterns for those environments compared to being in a gait analysis laboratory. However, there were still factors that could produce ”demand characteristics” (Rosenthal and Rosnow, 2009). These are changes in behavior due to expectations, whether conscious or not, of the purpose of the experiment or increased conscious control over normally unconscious movements.

Wearable motion capture provides a convenient and versatile means to record movement without instrumentation of the space, but it also is sensitive to challenges in calibration, placement of markers, and precision of recording. There are degrees of freedom with less range of motion that are nonetheless important biomechanically, such as knee and ankle frontal plane, that are measured with less validity than gold-standard marker-based tracking systems.

This analysis does not include statistical significance testing. We have calculated the common complexity measures and reported their mean and standard deviations where appropriate, or other commonly used reports such as percent variance explained in Figure 4A. The large differences or similarities are apparent to see the performance of these measures, but a more formal treatment could include statistical significance testing.

## 6 Conclusion

In this manuscript, we examine the complexity of different human locomotion activities using various measures of complexity pertaining to dimensionality, variability and nonlinear dynamics. We find that most of the measures rank the most commonly analyzed activity, walking forward in a straight line, to be the least complex. More importantly, different measures disagree about the relative complexity of the remaining activities. Thus, defining complexity as a single notion is challenging and we might need to be cognizant of what aspect of the data we wish to analyze when using any particular measure.

## Data Availability

The raw data supporting the conclusions of this article will be made available by the authors, without undue reservation.
